# Integrating qualitative and quantitative MRI analysis for optimizing DBS candidate selection in patients with disorders of consciousness

**DOI:** 10.3389/fneur.2025.1629319

**Published:** 2025-08-28

**Authors:** Marina Raguž, Petar Marčinković, Hana Chudy, Valentina Galkowski, Maja Majdak, Darko Orešković, Darko Chudy

**Affiliations:** ^1^Department of Neurosurgery, Dubrava University Hospital, Zagreb, Croatia; ^2^Catholic University of Croatia, Zagreb, Croatia; ^3^Department of Neurology, Dubrava University Hospital, Zagreb, Croatia; ^4^Department of Radiology and Interventional Radiology, Dubrava University Hospital, Zagreb, Croatia; ^5^Department of Surgery, School of Medicine, University of Zagreb, Zagreb, Croatia

**Keywords:** disorders of consciousness, MRI, qualitative analysis, morphometric analysis, deep brain stimulation, candidate selection

## Abstract

**Introduction:**

Disorders of consciousness (DoC) encompass a spectrum of clinical conditions with often indistinct boundaries, making accurate diagnosis and therapeutic decision-making particularly challenging. While advanced imaging techniques such as fMRI and PET reduce misdiagnosis risk, their limited availability in routine clinical settings underscores the need for alternative approaches. This study investigates whether the integration of qualitative and quantitative parameters derived from conventional MRI can improve diagnostic precision and support more accurate deep brain stimulation (DBS) candidate selection in DoC patients.

**Methods:**

Fifty consecutive DoC patients underwent comprehensive clinical, neurophysiological, and MRI assessment. Based on an integrated assessment of these findings, patients were classified as DBS candidates or non-candidates. MRI scans were qualitatively assessed for cortical and subcortical atrophy (including diffuse cortical, thalamic, and brainstem degeneration), ventricular enlargement, sulcal widening, leukoaraiosis, corpus callosum damage, gray-white matter border effacement, and extensive lesions (e.g., global ischemia or porencephalic cavities). Quantitative volumetric analysis was performed using the FreeSurfer pipeline.

**Results:**

Qualitative features such as leukoaraiosis, thalamic and cortical atrophy, ventricular enlargement, and corpus callosum lesions were significantly associated with DBS candidacy. Quantitative predictors included striatal volume, total gray matter, ventricular volume, CSF, and supratentorial volume. A combined model incorporating both qualitative and quantitative MRI data achieved high predictive accuracy (*AUC = 0.88*) for DBS candidacy.

**Conclusion:**

Integrating conventional MRI-based qualitative and quantitative assessments with clinical and neurophysiological evaluation may substantially improve DBS candidate selection in DoC patients, especially where functional imaging is unavailable. These findings support the development of practical MRI-based decision frameworks and call for multicenter validation. Despite increasing research on imaging and neuromodulation in DoC, studies directly comparing qualitative and quantitative structural MRI in the context of DBS candidacy remain scarce, highlighting a critical gap in the field.

## Introduction

Disorders of consciousness (DoC) are a group of conditions characterized by disruption or complete cessation of awareness and wakefulness (1, 2). These disorders encompass a clinical continuum including coma, vegetative state/unresponsive wakefulness syndrome (VS/UWS), and minimally conscious state (MCS) (1–5). Accurate assessment of patients with DoC is essential, as diagnostic accuracy directly impacts treatment decisions and outcomes. Standardized neurobehavioral tools - most notably the Coma Recovery Scale-Revised (CRS-R) - are widely used because they are sensitive in detecting signs of MCS and easy to apply in clinical settings (2, 6). Despite their utility, misdiagnosis rates can reach up to 40%, often due to the subjective nature of clinical observations and the difficulty in distinguishing reflexive from purposeful behavior (1, 5, 7). Moreover, the absence of behavioral responses does not necessarily indicate a lack of awareness; cognitive motor dissociation and sensory deficits can conceal residual consciousness (2). These limitations underscore the importance of complementary diagnostic tools. Neurophysiological techniques, including electroencephalography (EEG), evoked potentials, magnetoencephalography (MEG), and stimulus-induced EEG responses, have shown promise in enhancing diagnostic accuracy (8–12). Likewise, neuroimaging has become an indispensable element in the assessment of DoC, contributing both to diagnosis and prognostication. Functional modalities such as resting-state and task-based functional MRI (fMRI), diffusion tensor imaging (DTI), and positron emission tomography (PET) provide critical insights into cerebral function and structural integrity (13–15). These techniques not only enhance diagnostic accuracy but also help to clarify the nosology of DoC, contributing to a more refined conceptual framework for consciousness disorders. Recognizing their importance, several neurological societies have endorsed the use of neuroimaging as a valuable tool in diagnosing DoC (16). Notably, 18F-fluorodeoxyglucose PET has demonstrated the highest sensitivity among these, with approximately 85% congruence between PET findings and CRS-R classifications, particularly in identifying MCS patients (14, 17).

While these techniques are valuable, they are not always feasible in routine practice due to cost, limited availability, and technical complexity. Conventional structural MRI, in contrast, is widely accessible and can provide valuable information about brain integrity. Previous studies have shown that even without functional data, conventional MRI can help differentiate between VS/UWS and MCS through visual inspection of structural abnormalities (18, 19). Volumetric MRI analysis, using tools like the FreeSurfer pipeline, allows for automated, observer-independent evaluation of gray and white matter volumes, offering an objective supplement to clinical judgment (20). Structural MRI, therefore, holds promise not only in diagnosis but also in guiding patient selection for advanced interventions such as DBS. Invasive neuromodulatory interventions such as deep brain stimulation (DBS) are increasingly considered in selected DoC patients, particularly those in MCS (21, 22). DBS can target specific thalamic and brainstem nuclei, including the centromedian-parafascicular complex and reticular formation, which are involved in consciousness regulation (9, 12, 23). Accurate differentiation between UWS and MCS is therefore critical not only for prognosis but also for determining candidacy for such therapeutic interventions. Numerous studies have demonstrated that patients in an MCS are significantly more likely to respond favorably to DBS and other neuromodulatory treatments, likely due to the preservation of functional brain networks required for consciousness recovery (24, 25). Therefore, precise identification of MCS is a crucial step in optimizing therapeutic outcomes. However, current criteria for selecting DBS candidates are not standardized and often rely on coarse clinical impressions and limited imaging markers. Previous work from our group has focused on qualitative neuroradiological criteria for assessing DBS candidacy, emphasizing major structural lesions in key regions such as the brainstem, thalamus, and diencephalon (9, 12, 26). While informative, such assessments are inherently limited by their subjective nature and lack of quantitative precision.

Despite increasing evidence supporting the potential of DBS in select patients with DoC, the field still lacks standardized, scalable tools to guide patient selection. Current approaches often rely on functional neuroimaging such as PET or fMRI to detect covert consciousness, but these techniques are costly, technically demanding, and limited to highly specialized centers. Recent advances in quantitative structural MRI, including volumetric analyses and machine learning approaches, have demonstrated strong potential for outcome prediction and stratification in DoC using conventional imaging sequences (27, 28). In parallel, expert reviews and guideline initiatives by the European Academy of Neurology and the Coma Science Group have emphasized the need for integrative, multimodal strategies that incorporate structural imaging into the clinical workflow (29, 30). Additionally, recent findings suggest that structural brain integrity, as revealed by MRI, may play a critical role in predicting response to DBS and guiding surgical candidacy (31).

To address this gap, the present study proposes a novel framework that integrates qualitative structural MRI markers with quantitative volumetric metrics to emulate real-world DBS selection processes. By leveraging widely available MRI modalities, our approach aims to support standardized, evidence-informed decision-making in diverse clinical settings, including those without access to functional imaging.

## Methods

### Patients

This prospective observational study included patients admitted to the Department of Neurosurgery, Referral Centre for Stereotactic and Functional Neurosurgery, Dubrava University Hospital, Zagreb, Croatia, between July 1^st^, 2021, and September 30^th^, 2024. The primary objective was to assess candidacy for DBS as a therapeutic option for restoring consciousness in patients with DoC. A total of 50 consecutive DoC patients were enrolled, regardless of the underlying etiology or duration of their condition. All patients underwent a comprehensive diagnostic workup during the observation period. Neurophysiological assessments included somatosensory, motor, and brainstem auditory evoked potentials, as well as 12- or 24-h EEG monitoring. Clinical evaluation was performed using standardized rating scales, including the Coma/Near Coma (C/NC) scale, the Rappaport Disability Rating (RDR) scale, and the CRS-R, with the addition of neuroimaging analysis (9, 12, 26). Based on this multimodal evaluation, DBS candidacy included preserved long-latency evoked potentials, stimulus-induced EEG reactivity, and the absence of bilateral brainstem dysfunction (9, 12, 26).

**Figure 1:**
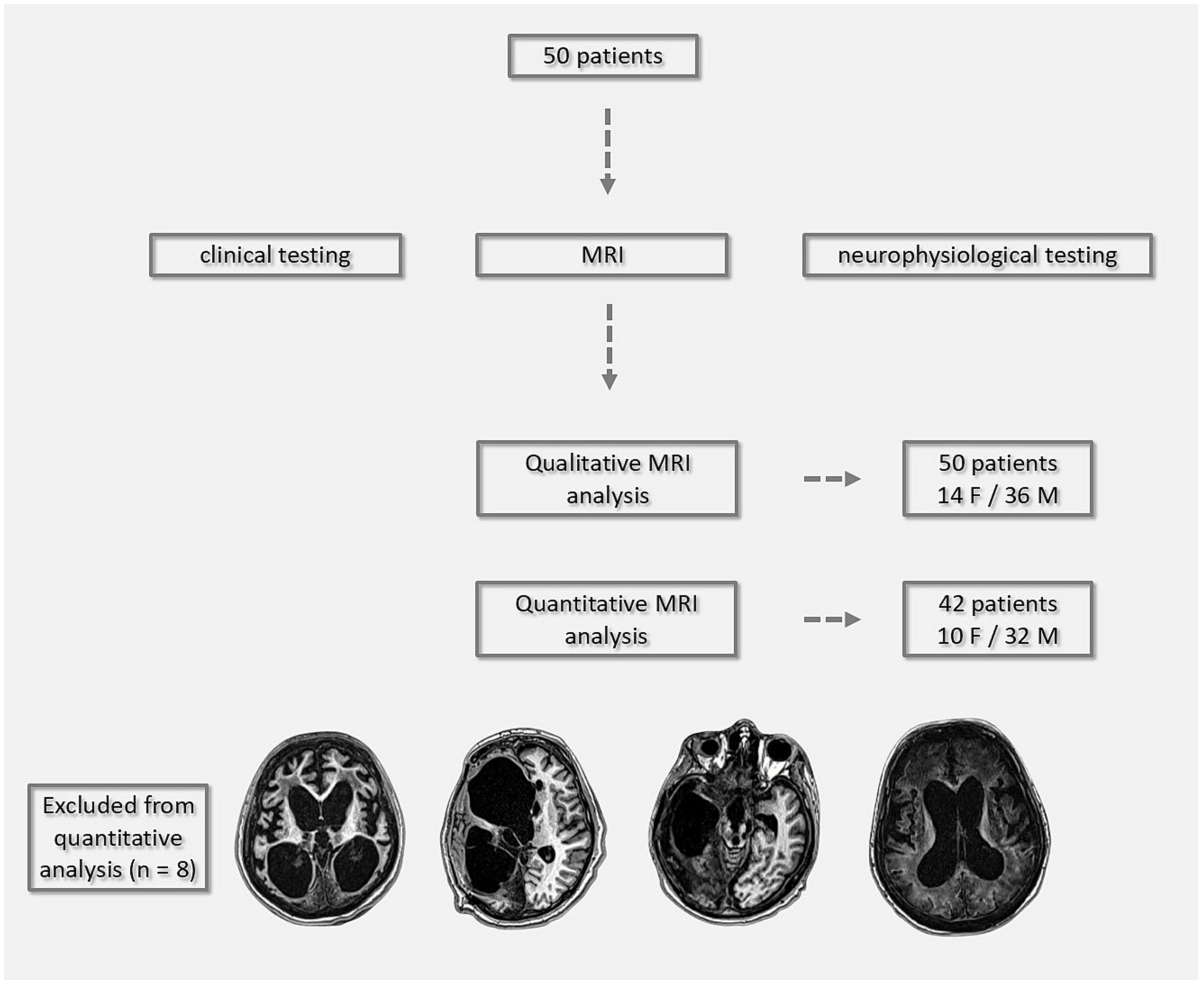
Flowchart of the assessment protocol for 50 patients with DoC. All patients underwent clinical testing, neurophysiological evaluations, and MRI. Qualitative MRI analysis was performed in the full cohort (14 females, 36 males). Quantitative MRI analysis was feasible in 42 patients (10 females, 32 males); eight patients were excluded due to extensive structural damage or large ischemic lesions that precluded automated morphometric processing, with representative MRI scans shown below.

Inclusion criteria were based on established diagnostic definitions of DoC (13), with additional requirements that patients have stable hemodynamic and respiratory function, no contraindications for MRI, and a minimum DoC duration of six weeks. All 50 patients underwent qualitative MRI analysis, while quantitative morphometric analysis was feasible in 42 patients; eight were excluded due to extensive structural brain damage or large ischemic lesions that interfered with automated morphometric processing (see Figure 1 for assessment protocol and representative MRI exclusions). A review of demographic and clinical characteristics of the excluded patients revealed no meaningful differences compared to the included group in terms of age, sex, diagnosis, reducing concern for selection bias.

This study was conducted following the ethical standards of the Dubrava University Hospital Ethics Board and the Declaration of Helsinki. Written informed consent was obtained from all participants or their legal representatives. The study protocol was approved by the Institutional Review Board of Dubrava University Hospital, Zagreb, Croatia (reference number: 2020/2409–02).

### MRI acquisition

All MRI scans were performed on a 1.5 T scanner (MAGNETOM Aera, Siemens Healthineers, Erlangen, Germany) using a 24-channel head coil. Standard clinical sequences were acquired for qualitative evaluation, alongside a high-resolution three-dimensional T1-weighted magnetization-prepared rapid gradient echo (MPRAGE) sequence for quantitative volumetric analysis.

Scanning parameters were as follows: repetition time (TR) = 2,400 ms, echo time (TE) = 3 ms, flip angle = 8°, matrix size = 256 × 232, field of view (FOV) = 218 × 240 mm, voxel size = 1.0 × 1.0 × 1.0 mm, slice thickness = 1.0 mm, total of 160 slices, and acquisition time of 5 min and 5 s.

All patients underwent MRI under sedation, with continuous monitoring of vital signs, including oxygen saturation and heart rate, performed by trained medical staff to ensure safety throughout the procedure.

### Qualitative analysis

Qualitative MRI analysis was performed independently by two experienced observers. According to literature, the evaluation included the following key features: diffuse cortical atrophy (DCA), brainstem/thalamus (BS/T) atrophy or degeneration, corpus callosum (CC) lesions and atrophy, ventricular enlargement (VE), sulcal widening (SW), leukoaraiosis, with addition of gray-white matter (GM/WM) border effacement and major focal lesions (e.g., global ischemia, porencephalic cavities) (18).

**Figure 2:**
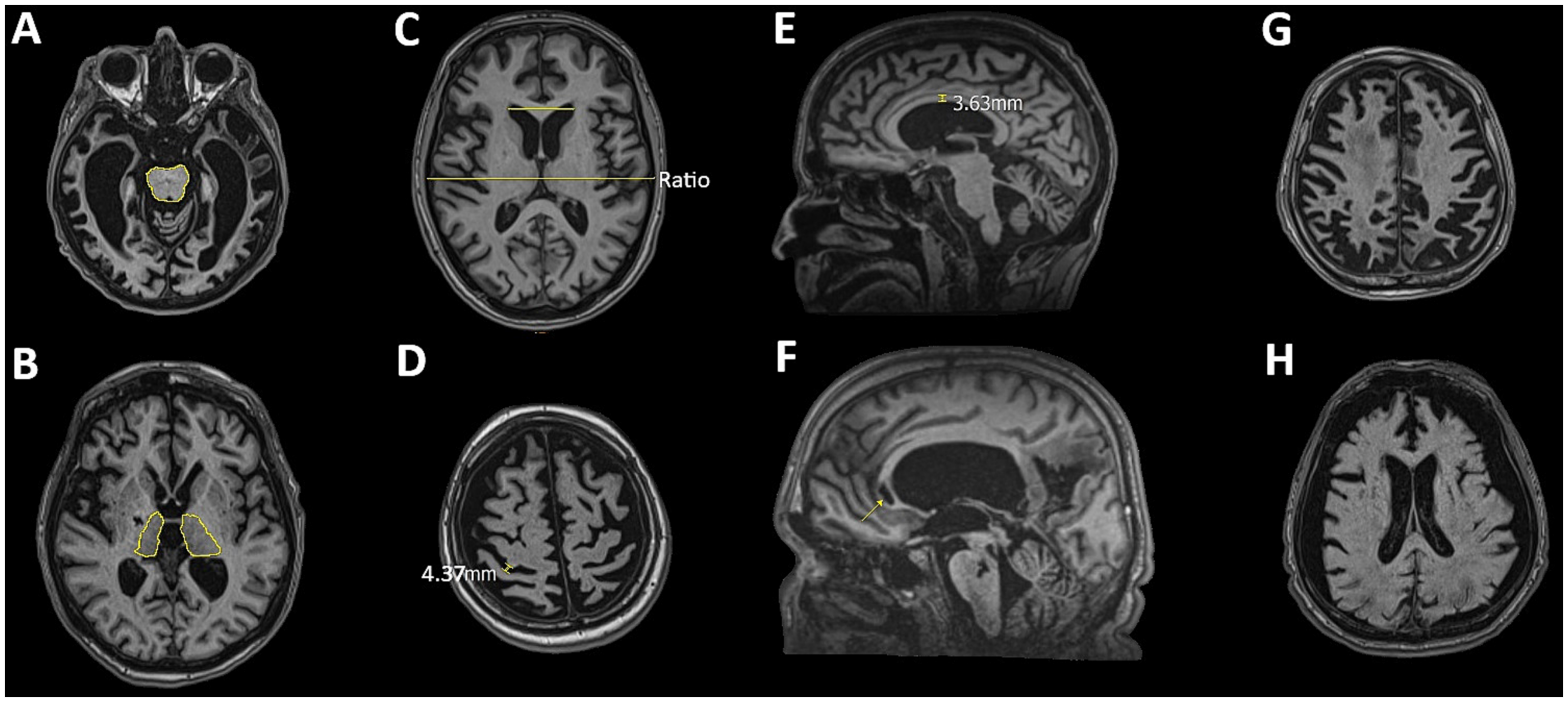
Representative MRI slices illustrating features assessed in the qualitative analysis: **(A)** brainstem atrophy, **(B)** thalamic atrophy, **(C)** ventricular enlargement assessed using the Evans Index, **(D)** sulcal widening, **(E)** corpus callosum thinning (degeneration), **(F)** corpus callosum lesions, **(G)** diffuse cortical atrophy, and **(H)** effacement of the gray-white matter border.

DCA, BS/T atrophy, and CC lesions were assessed categorically (presence/absence). VE was quantified using the Evans Index (EI), with moderate VE defined as EI = 0.31–0.74 and severe VE as EI > 0.74. SW was evaluated at the level of the central sulcus. Moderate enlargement was defined as subarachnoid space dilation ≤ 0.4 cm; values > 0.4 cm were classified as severe. Leukoaraiosis was graded based on the presence of periventricular caps, smooth rings, and confluent changes: moderate if limited to periventricular zones, and severe if extending into deep and subcortical white matter. CC atrophy was measured at the midsagittal plane. Thickness between 0.2–0.4 cm was classified as moderate atrophy; values < 0.2 cm were considered severe (18). The analysis also noted the effacement or blurring of the GM/WM border and multiple lesions in the BS and thalamus. Major lesions—including global ischemia, large porencephalic cavities, and extensive damage affecting ≥30% of lobar or basal ganglia volume—were used as exclusion criteria for quantitative analysis (32). BS and thalamic atrophy were evaluated at multiple anatomical levels to ensure comprehensive coverage (see Figures 1, 2).

In cases of inter-observer disagreement, a consensus was reached via joint re-evaluation. Inter-rater reliability was 90% (Cohen’s *κ* = 0.82), and intra-rater reliability was 92% (Cohen’s *κ* = 0.85), indicating a high level of agreement in qualitative assessments.

### Quantitative analysis

Quantitative volumetric analysis of brain structures and compartments was performed using the FreeSurfer image analysis suite (version 7.4.1; https://surfer.nmr.mgh.harvard.edu). The processing pipeline included several key steps: intensity normalization, motion correction, and removal of non-brain tissue using a hybrid watershed/surface-based approach. Images were then transformed into Talairach space and subjected to automated segmentation of subcortical white matter (WM) and deep gray matter (GM) structures, following validated procedures (33, 34). FreeSurfer was used to differentiate GM, WM, and cerebrospinal fluid (CSF), providing reliable and anatomically accurate volumetric measurements of brain compartments. All datasets underwent rigorous quality control to ensure segmentation fidelity. Each T1-weighted MRI scan was visually inspected using the FreeView tool, which allowed for overlay of segmentation and parcellation maps on the original images. This step enabled the detection of motion artifacts or segmentation errors. Any inconsistencies were corrected before final volumetric data were included in the analysis. All segmentations were visually inspected by two independent raters; in cases of gross misclassification due to lesions, minor manual edits were performed following FreeSurfer guidelines. Corrections were primarily limited to subcortical boundaries and regions near large lesions and were kept minimal to ensure reproducibility.

### Statistical analysis

All statistical analyses were performed using MedCalc Statistical Software (version 12.5.0; MedCalc Software, Ostend, Belgium; https://www.medcalc.org). Results are presented in tables and figures, with continuous variables expressed as mean ± standard deviation. The distribution of continuous variables was assessed using the Kolmogorov–Smirnov test. Depending on normality, associations between parameters were evaluated using either the Pearson correlation coefficient or the Spearman rank correlation test. Group comparisons were performed using either the Student’s t-test or the Mann–Whitney U test, as appropriate. Analysis of covariance (ANCOVA) was conducted to adjust for covariates, accompanied by Levene’s test for equality of variances. Bonferroni correction was applied for multiple comparisons. To control for potential confounders such as age and time since injury, brain volume measurements were adjusted using a multiple linear regression model. For classification purposes, binary logistic regression was used to identify predictors of DBS candidacy. Receiver operating characteristic (ROC) curve analysis was performed to assess the diagnostic performance of selected parameters. A *p*-value < 0.05 was considered statistically significant. A formal *a priori* power analysis was not conducted due to the exploratory nature of the study. However, the final sample size (*n* = 50) is consistent with or larger than those used in previous structural MRI and DBS studies in patients with DoC, and was deemed adequate to support the statistical analyses performed.

## Results

This study included 50 consecutive patients with DoC, of whom 14 (28%) were female and 36 (72%) male. The overall mean age was 41.84 ± 16.51 years (range 4–81). Female patients had a mean age of 53.0 ± 12.99 years (range 26–81), while male patients had a mean age of 37.92 ± 15.93 years (range 4–64). At initial assessment, the majority of patients (48/50; 96%) were in a UWS, and only 2 (4%) were classified as being in an MCS. Out of the total cohort, 23 patients met previously defined criteria for DBS candidacy. The mean age of DBS candidates was 41.04 ± 16.29 years (range 16–64), while non-candidates (*n* = 27) had a mean age of 42.52 ± 16.97 years (range 4–81). Regarding etiology, 32 patients presented with hypoxic–ischemic brain injury (HI), most commonly following cardiac arrest or intracerebral hemorrhage, while 18 patients sustained traumatic brain injury (TBI). Detailed demographic and clinical characteristics of the two subgroups are presented in Table 1.

**Table 1:** Demographic details of patients included in the study.

	Total n (HI/TBI)	Age (years) (mean ± SD)	DoC duration (months) (mean ± SD)	Sex (F/M)	DOC status (UWS/MCS)
DBS candidates	23 (15/8)	41.04 ± 16.29 (16–64)	6 ± 7.84 (1–27)	3/20	21/2
DBS non-candidates	27 (18/9)	42.52 ± 16.97 (4–81)	7 ± 9.69 (2–37)	11/16	27/0

DBS, deep brain stimulation; HI, hypoxic–ischemic injury; TBI, traumatic brain injury; F, female; M, male; DoC, disorder of consciousness; UWS, unresponsive wakefulness syndrome; MCS, minimally conscious state.

### Results of qualitative analysis

Qualitative analysis revealed several significant correlations between structural MRI parameters and the initial clinical state of patients with DoC. The strongest negative correlation was observed for BS/T atrophy (*ρ* = −0.48, *p* = 0.0005), followed by DCA (*ρ* = −0.38, *p* = 0.007). GM/WM border effacement showed a marginal significance (*ρ* = −0.25, *p* = 0.07).

In the subgroup of patients identified as candidates for DBS, significant associations were observed with the presence of the CC lesions (*ρ* = −0.31, *p* = 0.03), DCA (*ρ* = −0.27, *p* = 0.05), and leukoaraiosis (*ρ* = −0.43, *p* = 0.001), as well as thalamic atrophy (*ρ* = −0.33, *p* = 0.02). A trend toward significance was again observed for GM/WM effacement (*ρ* = −0.26, *p* = 0.06).

**Figure 3:**
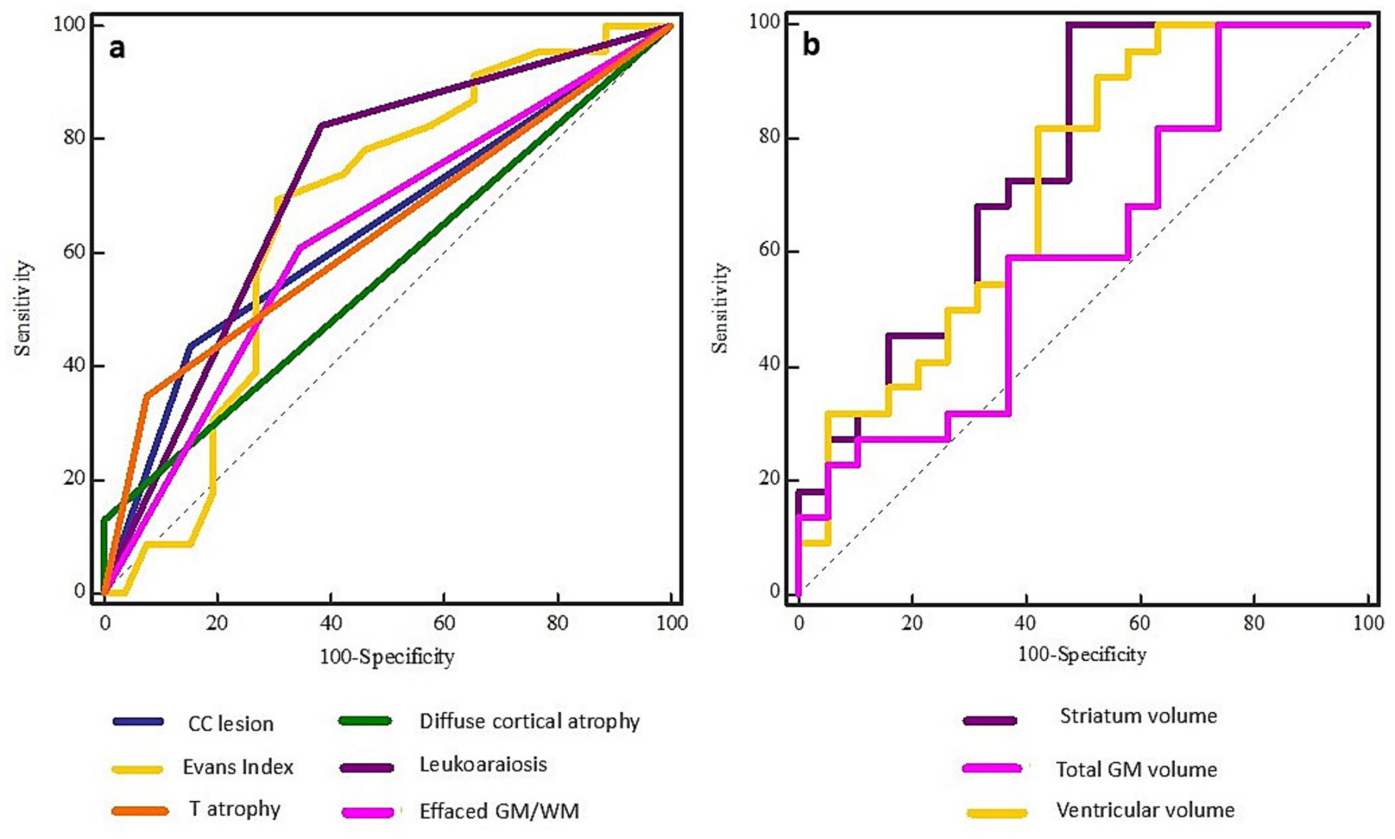
ROC curves for qualitative (left, **A**) and quantitative (right, **B**) MRI features in predicting DBS candidacy in patients with DoC. Among qualitative parameters, leukoaraiosis demonstrated the highest predictive accuracy (*AUC* = 0.72), followed by Evans Index and corpus callosum lesions. In the quantitative domain, striatum volume showed the strongest predictive value (*AUC* = 0.75), with ventricular volume, gray matter volume, and supratentorial volume also contributing substantially.

To further assess the diagnostic utility of these qualitative features in predicting DBS candidacy, a ROC analysis was performed (Figure 3A). Leukoaraiosis demonstrated the highest predictive value (Area Under Curve, *AUC* = 0.72, Sensitivity, SE = 82.61, Specificity, S*P* = 61.54, Youden index, *Y* = 0.44, *p* = 0.001). In contrast, CC lesions (*AUC* = 0.64, SE = 43.48%, S*P* = 84.62%, *Y* = 0.28, *p* = 0.03) and EI (*AUC* = 0.67, SE = 69.57%, S*P* = 69.23%, *Y* = 0.38, *p* = 0.03) demonstrated poor diagnostic value. Although thalamic atrophy (*AUC* = 0.63, SE = 34.78%, S*P* = 92.31%, *Y* = 0.27, *p* = 0.01), GM/WM effacement (*AUC* = 0.63, SE = 60.87%, S*P* = 65.38%, *Y* = 0.26, *p* = 0.06), and DCA (*AUC* = 0.56, SE = 13.04%, S*P* = 100.00%, *Y* = 0.13, *p* = 0.06) showed moderate diagnostic potential, their overall predictive strength was lower compared to leukoaraiosis.

### Results of quantitative analysis

Quantitative analysis included 42 patients; eight patients were excluded due to extensive structural brain damage or segmentation failure (see Methods for details). Quantitative volumetric analysis, adjusted for age and time since injury, revealed several significant associations between brain volumes and the initial clinical status of patients with DoC. Specifically, CC volume (*ρ* = 0.28, *p* = 0.04) and total GM volume (*ρ* = 0.29, *p* = 0.05) showed statistically significant positive correlations with the initial DoC state.

In the subgroup of DBS candidates, several brain regions demonstrated significant correlations, namely total brain volume (*ρ* = 0.32, *p* = 0.03), total GM volume (*ρ* = 0.36, *p* = 0.04), CSF volume (*ρ* = −0.41, *p* = 0.003), supratentorial volume (*ρ* = 0.36, *p* = 0.02), CC (*ρ* = 0.29, *p* = 0.04), thalamic (*ρ* = 0.32, *p* = 0.03), striatum (*ρ* = 0.55, *p* = 0.0002), and ventricular volume (*ρ* = −0.40, *p* = 0.008). Brainstem and cerebellar white matter volumes showed borderline significance (*p* ≈ 0.05).

To assess their predictive value for DBS candidacy, ROC analysis was conducted (Figure 3B). The following volumes demonstrated the strongest diagnostic performance: striatum volume (*AUC* = 0.75, *SE* = 100.00, *SP* = 52.63, *Y* = 0.53, *p* = 0.001), ventricular volume (*AUC* = 0.73, *SE* = 81.82, *SP* = 60.00, *Y* = 0.42, *p* = 0.0004) and total GM volume (*AUC* = 0.72, *SE* = 86.36, *SP* = 55.00, *Y* = 0.41, *p* = 0.008), indicating fair diagnostic utility in candidate selection. Supratentorial volume (*AUC* = 0.71, *SE* = 72.73, *SP* = 70.00, *Y* = 0.43, *p* = 0.01) and CSF volume (*AUC* = 0.71, *SE* = 90.91, *SP* = 52.17, *Y* = 0.43, *p* = 0.01) also emerged as relevant predictors with notable accuracy. Poor predictive value was observed for total brain volume (*AUC* = 0.68, *SE* = 63.64, *SP* = 80.00, *Y* = 0.44, *p* = 0.03) and thalamic volume (*AUC* = 0.69, *SE* = 95.45, *SP* = 52.63, *Y* = 0.48, *p* = 0.03), highlighting their utility in guiding the decision-making process for DBS candidacy. On the other hand, parameters like cerebellar WM (*AUC* = 0.67, *SE* = 68.18, *SP* = 70.00, *Y* = 0.38, *p* = 0.05) and CC volume (*AUC* = 0.66, *SE* = 90.91, *SP* = 42.31, *Y* = 0.33, *p* = 0.04) demonstrated lower discriminative power, yet may still contribute to the overall assessment strategy.

### Results of multivariate and comparative analyses

To enhance the selection process for DBS candidates, we applied a binary logistic regression model that integrated key predictors from both qualitative and quantitative MRI assessments. The model incorporated qualitative features such as leukoaraiosis, along with quantitative volumetric parameters including GM, ventricular, and striatal volumes. This combined approach significantly improved classification accuracy, yielding a high predictive value (*AUC* = 0.88, 95% CI 0.74–0.96, *p* = 0.0005).

**Figure 4:**
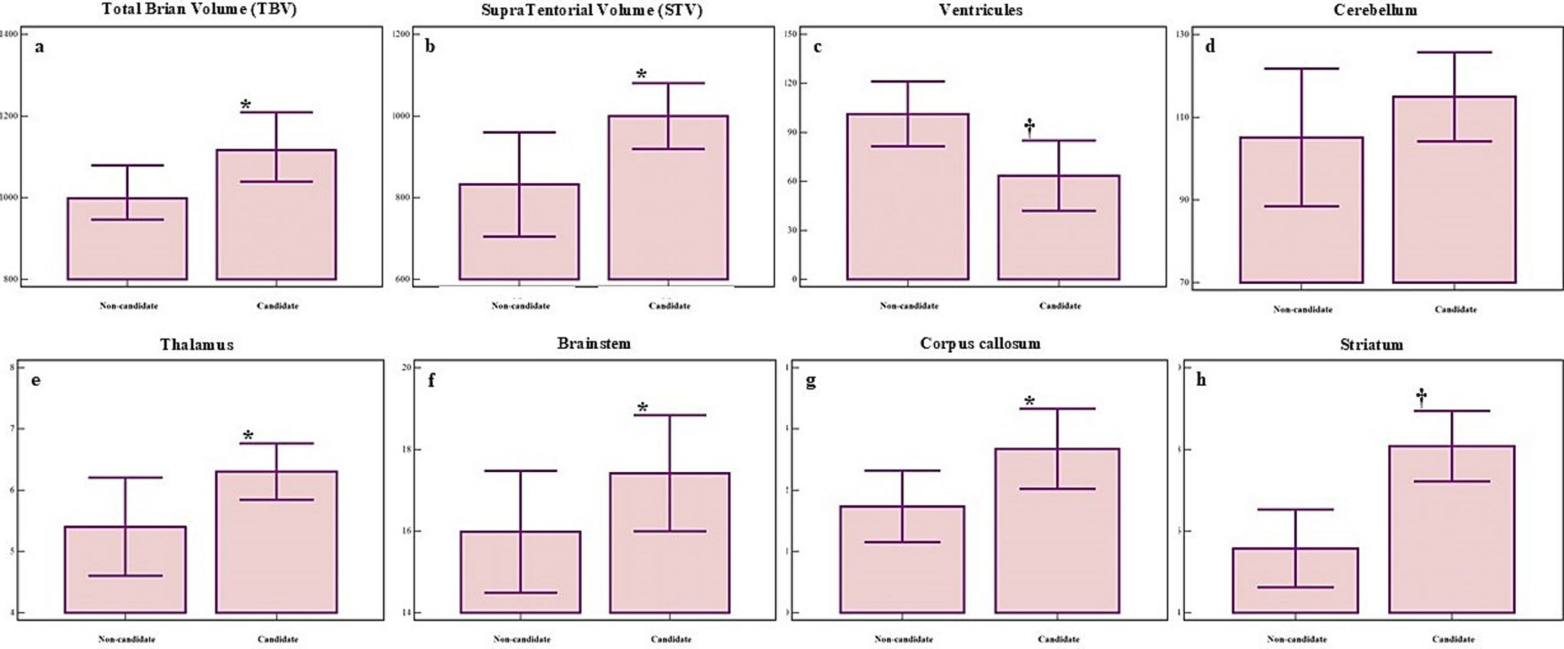
Significant morphometric differences in several brain regions between non-candidates (left bar) and candidates (right bar) for DBS. Panels show volumetric differences for: **(A)** total brain volume (BV), **(B)** supratentorial volume (STV), **(C)** ventricular volume, **(D)** cerebellar volume, **(E)** thalamic volume, **(F)** brainstem volume (BS), **(G)** corpus callosum volume (CC), **(H)** striatum volume. Group means ± standard deviations are shown. *p* ≤ 0.05 marked with *, *p* ≤ 0.01 marked with †.

In addition to regression modeling, comparative analyses revealed significant morphometric differences between DBS candidates and non-candidates (Figure 4). Group comparisons identified significant differences in total brain volume (*t* = 2.18, *p* = 0.03), supratentorial (*t* = 2.36, *p* = 0.02), and ventricular volume (*t* = 2.69, *p* = 0.01). Similarly, notable differences were observed in the thalamic (*t* = 2.11, *p* = 0.04), CC volume (*U* = 191.00, *p* = 0.04), and Evans index values (*t* = 2.10, *p* = 0.04). Highly relevant changes were also identified in the BS (*U* = 242.00, *p* = 0.05), leukoaraiosis (*U* = 166.50, *p* = 0.003), DCA (*U* = 260.00, *p* = 0.05), and striatum (*U* = 75.5, *p* = 0.0005). These findings underscore the importance of combining robust statistical models with detailed morphometric evaluations to optimize the selection process.

To further explore the impact of candidate status on structural features, we performed an ANCOVA using gender, age, and time since injury as covariates. Significant group effects were found for several qualitative features, including DCA [*F* (1, 35) = 11.86, *p* = 0.001], CC lesion [*F* (1, 35) = 18.16, *p* < 0.001], leukoaraiosis [*F* (1, 35) = 5.66, *p* = 0.02], BS [*F* (1, 35) = 12.77, *p* = 0.001] and thalamic atrophy [*F* (1, 35) =  29.01, *p* < 0.001]. Additionally, significant differences were observed in several quantitatively assessed measures, including cerebral WM [F (1, 36) = 8.33, *p* = 0.006], total GM [*F* (1, 36) = 4.09, *p* = 0.05], CSF [*F* (1, 36) = 9.32, *p* = 0.004], striatum [*F* (1, 36) = 10.52, *p* = 0.02], brainstem [*F* (1, 36) = 12.77, *p* = 0.001] and thalamic volume [*F* (1, 36) = 7.29, *p* = 0.01]. These results highlight distinct morphometric and structural differences between DBS candidates and non-candidates, particularly within GM and WM structures, CSF compartments, and key regulatory hubs such as the striatum, thalamus, and brainstem.

## Discussion

To the best of our knowledge, this is the first study to investigate the association between qualitatively assessed structural brain abnormalities and quantitatively measured brain compartment volumes using an automated, observer-independent volumetric MRI approach in patients with DoC. All patients underwent comprehensive clinical, neurophysiological, and neuroradiological evaluation and were subsequently classified into two subgroups - DBS candidates and non-candidates - based on integrative criteria. Our primary aim was to identify structural brain markers that could aid in determining DBS candidacy and to highlight the potential of MRI-based volumetric analysis in improving patient selection and therapeutic outcomes. Results from the qualitative analysis demonstrated that BS and thalamic atrophy, DCA, and effacement of the GM/WM border were significantly associated with the initial clinical state (i.e., UWS or MCS). Among structural features, leukoaraiosis and CC lesions emerged as the strongest predictors of DBS candidacy, while thalamic atrophy, DCA, and GM/WM border effacement showed moderate but clinically relevant diagnostic value.

The brainstem and thalamus play central roles in regulating consciousness, arousal, and vital autonomic functions, as consistently emphasized in prior research (37–39). Brainstem atrophy has been associated with impaired consciousness and autonomic dysregulation, serving as a structural marker of poor neurological outcomes in patients with prolonged UWS or MCS (19, 40). In parallel, thalamic atrophy compromises the integration of sensory and motor information, contributing to deficits in awareness, cognition, and behavioral responsiveness (36, 39, 41). Both brainstem and thalamic degeneration have been strongly correlated with DoC severity, lower CRS-R scores, and limited recovery potential (19, 42–47), reinforcing their integral roles within the broader neural network of consciousness (35). A plausible neuropathological explanation for thalamic atrophy in DoC is axonal degeneration, manifesting as reduced fiber density within thalamic nuclei and disruption of thalamocortical connectivity (48). Structural changes such as anterior nucleus shrinkage and dorsal posterior nucleus hypertrophy may further impair large-scale integration and information flow, exacerbating clinical presentation (48). Conversely, brainstem lesions, particularly those involving the tegmentum, are highly specific for DoC. Damage to nuclei such as the rostral raphe complex and locus coeruleus can induce profound alterations in consciousness and, in severe cases, fatal dysregulation of vital functions (49, 50). These findings highlight the centrality of the ascending arousal system and its vulnerability in severe brain injury.

In addition, the observed relationship between cortical atrophy and diminished recovery potential suggests that the extent and distribution of cortical damage may serve as an important marker when evaluating a patient’s eligibility for DBS. Atrophy in specific regions, particularly within the frontal and parietal lobes, has been associated with reduced neuroplasticity and a limited capacity to respond to therapeutic stimulation (51–54). Notably, cortical thinning is most pronounced in the frontal and temporal regions, as well as bilaterally within the thalamus, especially during the early post-traumatic phase, typically within the first three months following TBI, leading to DoC (55). Moreover, patients with post-TBI DoC often exhibit a pattern of generalized cerebral atrophy, accompanied by focal volume loss in both gray and white matter structures. These include the cerebellum, thalamus, and hippocampus in the gray matter, as well as the brainstem, corpus callosum, and corona radiata in the white matter (56). These structural deficits are particularly relevant in the context of DBS, as neurophysiological assessments suggest that individuals with widespread cortical atrophy demonstrate lower potential for motor or cognitive recovery (57). Such findings reinforce the notion that cortical integrity remains a critical determinant in selecting appropriate candidates for invasive neuromodulatory interventions.

Furthermore, although less frequently studied, GM/WM border effacement is increasingly recognized as a relevant marker of neurodegeneration. The blurring or loss of this anatomical distinction has been reported in conditions such as Alzheimer’s disease and advanced TBI, where it reflects severe structural compromise, cortical thinning, and a diminished capacity for neuroplasticity (58, 59). Disruption of the GM/WM interface likely reflects a breakdown in the organizational integrity of large-scale neural networks that underlie cognition and arousal, thereby compromising both consciousness and the potential for recovery (60). Our findings suggest that GM/WM border effacement in patients with DoC may reflect more extensive structural disorganization and reduced neuroplastic potential. Although we did not evaluate clinical outcomes, this feature has been previously linked to diminished recovery potential in other neurodegenerative and traumatic conditions (40, 61).

This pattern is reminiscent of changes observed in other neurodegenerative diseases and likely involves damage to structural pathways critical for adaptive reorganization and functional recovery (62). Its presence in our cohort highlights its potential as an imaging correlate of severe neural compromise. Further research is needed to clarify its prognostic value and pathophysiological significance in DoC.

Interestingly, the significant correlations between brainstem atrophy, diffuse cortical atrophy, and GM/WM border effacement suggest that these phenomena may not be isolated, but rather interconnected elements of a progressive neurodegenerative cascade. In this framework, effacement of the GM/WM border could reflect downstream consequences of early subcortical degeneration, particularly involving the brainstem and thalamus, thus offering insight into the structural evolution of consciousness disorders. Given the lack of standardized rating criteria for GM/WM border effacement, future studies should aim to establish validated scales or automated tools to enhance inter-rater reliability and clinical implementation.

In our study, leukoaraiosis and corpus callosum lesions emerged as the strongest qualitative predictors for identifying appropriate DBS candidates among DoC patients. Leukoaraiosis, reflecting chronic microvascular pathology, has been shown to disrupt critical large-scale brain networks, including the default mode and salience networks, which are essential for maintaining consciousness and enabling cognitive integration. This disruption may significantly hinder cognitive and functional recovery in DoC patients (63). Moreover, leukoaraiosis has been identified as a key risk factor for post-stroke disturbances of consciousness, contributing to prolonged and often incomplete recovery trajectories (64). Pathophysiologically, leukoaraiosis is typically attributed to small vessel disease, which promotes chronic hypoxia, demyelination, and breakdown of the blood–brain barrier, leading to widespread white matter damage and impaired connectivity (65). These changes may underlie the reduced responsiveness to neuromodulatory interventions observed in affected patients. Similarly, the presence of corpus callosum lesions was strongly associated with poorer outcomes, particularly in patients diagnosed with unresponsive wakefulness syndrome. Our findings align with previous work showing that structural damage to the corpus callosum significantly reduces the likelihood of recovery in severe DoC (66). Given its role in interhemispheric communication and global integration, corpus callosum integrity appears critical for the re-emergence of conscious behavior and responsiveness. Its disruption may further isolate functional networks, limiting recovery potential even in structurally preserved cortical areas.

While qualitative imaging offers valuable insights, quantitative structural analysis provides a more objective and reproducible framework for evaluating DoC. Numerous studies have employed volumetric MRI techniques to improve diagnostic and prognostic accuracy across a range of neurodegenerative conditions (40, 67). However, in the context of DoC, such studies remain limited in number and scope. Existing research has predominantly focused on distinguishing between patients in UWS and MCS (20) or exploring the prognostic significance of isolated brain metrics (53). In a prior longitudinal morphometric study, our group demonstrated that DoC patients who responded positively to DBS exhibited volume increases in specific cortical and subcortical regions following treatment (26). More recently, a study investigating patients undergoing CM-pf DBS found that better-preserved gray matter, particularly within the striatum and cerebellum, was associated with improved levels of consciousness (68). These findings support the notion that structural brain integrity contributes to recovery potential and may reflect neuroplastic responsiveness to stimulation.

In our current analysis, several quantitative structural markers emerged as significant predictors of DBS candidacy. These included striatal and ventricular volumes, total GM and CSF volumes, as well as supratentorial and total brain volumes. Additionally, thalamic volume was a differentiator between groups. An especially noteworthy finding was the relevance of the total brain volume-to-intracranial volume ratio and GM volume-to-intracranial volume ratio, both of which were reduced in non-candidates. These results underscore brain atrophy as a potentially critical limiting factor in DBS eligibility. Among these, reduced striatal volume appeared particularly relevant, showing a consistent difference between DBS candidates and non-candidates. This may reflect the critical role of basal ganglia structures in supporting arousal and goal-directed behavior. While prior studies have often focused on cortical and thalamic atrophy, our findings suggest that striatal volumetry, derived from conventional structural MRI, could provide additional information for assessing DBS suitability in patients with DoC. Our findings align with previous work by Silva et al. (53), who identified GM volume as a key prognostic marker in survivors of cardiac arrest, emphasizing the role of preserved gray matter in enabling recovery. Similarly, Annen et al. (20) reported more severe degrees of brain atrophy in UWS patients compared to those in MCS, supporting the association between volumetric preservation and better outcomes. Neuropathological investigations of well-known UWS cases, such as Karen Ann Quinlan and Terri Schiavo, further highlight this relationship. Both cases revealed profound and widespread atrophy after years in a vegetative state (69–71). Interestingly, thalamic atrophy was more pronounced than cortical loss, reinforcing the thalamus’s role as a central hub for cognition, awareness, and arousal (69). These morphometric findings may not only inform DBS candidacy but also serve as potential biomarkers of neuroplastic potential, helping to predict responsiveness to neurorehabilitation strategies. In our cohort, patients classified as non-candidates exhibited significant atrophy of the brainstem, thalamus, corpus callosum, and striatum - regions functionally tied to arousal, interconnectivity, and goal-directed behavior. These results emphasize the diagnostic and prognostic relevance of quantitative MRI metrics in assessing DBS suitability and support the use of volumetric data as a clinically meaningful tool. Importantly, many of the qualitative features identified as relevant by our model, such as thalamic atrophy, ventricular enlargement, leukoaraiosis, and GM/WM border effacement, mirror the neuroanatomical structures routinely assessed by clinicians. Traditional MRI-based decisions often focus on excluding diffuse cortical destruction, confirming the preservation of subcortical hubs like the thalamus and brainstem, and identifying signs of irreversible damage such as corpus callosum lesions or extensive white matter disease. Our findings suggest that, rather than replacing clinical judgment, the model serves to formalize and quantify these expert-driven heuristics. This convergence reinforces the validity of current clinical approaches and highlights how automated volumetric analysis can enhance objectivity, reproducibility, and standardization in candidate selection. The distinct differences between DBS candidates and non-candidates, particularly in subcortical structures, also highlight a critical gap in the literature, underscoring the novelty and clinical relevance of our findings.

Several limitations of this study should be acknowledged. Although prospectively designed, the cohort included patients with considerable variability in terms of age, sex, etiology, and duration of DoC before evaluation, all of which may introduce clinical and structural heterogeneity. Moreover, the single-center design may limit the generalizability of the findings to other populations or institutional contexts with different clinical protocols and resource availability. While the inclusion of 50 patients represents a meaningful sample for this highly specialized population, larger multicenter studies will be needed to replicate and validate our findings and to enhance statistical power for stratified analyses. Additionally, although major clinical and imaging confounders were considered, other potential influences, such as comorbidities, medication history, socioeconomic factors, and access to rehabilitation, were not systematically controlled for and may have impacted brain structure or recovery potential. An important limitation is the absence of longitudinal outcome data at this stage. Although follow-up of this patient cohort is actively ongoing, our current findings are limited to baseline structural predictors and cannot yet speak to how these relate to long-term recovery or DBS responsiveness. Future analyses will address this gap by integrating follow-up outcomes into the predictive framework. Finally, although qualitative MRI ratings were performed independently by experienced raters and demonstrated high inter- and intra-rater reliability, the absence of a universally accepted scoring system for some features (e.g., GM/WM border effacement) may limit reproducibility across centers. Standardized imaging protocols and multicenter harmonization will be essential for translating these findings into broader clinical practice. These preliminary findings offer a basis for future validation efforts, ideally through harmonized multicenter studies aimed at establishing reproducible imaging-based criteria for DBS candidate selection.

## Conclusion

This study provides compelling evidence that integrating qualitative and quantitative structural MRI metrics offers a powerful framework for identifying candidates for DBS among patients with DoC. Importantly, our model does not predict treatment responsiveness or long-term clinical outcomes; rather, it seeks to emulate the current clinical framework for DBS candidate selection based on integrated neuroimaging and physiological evaluation. Striatal volume showed potential relevance in differentiating candidates from non-candidates, adding to the value of assessing subcortical structures alongside more commonly evaluated regions such as the thalamus and cortex. These findings suggest that subcortical volumetric profiling may offer additional support in DBS decision-making. While previous approaches have focused on either visual inspection or advanced functional imaging, our findings demonstrate that widely available conventional MRI, when rigorously analyzed, can deliver clinically actionable insights into brain integrity and recovery potential. This MRI-based framework can be deployed in routine clinical settings, even where functional modalities are unavailable, addressing a major gap in the standard of care. However, structural imaging should not be viewed in isolation. We emphasize that DBS candidacy must be guided by a multidisciplinary approach that integrates detailed clinical, neurophysiological, and neuroradiological assessments to fully capture the complex biology of consciousness disorders.

Looking ahead, emerging technologies, including AI-powered volumetric analysis, machine learning–based classification tools, and brain–computer interfaces, are poised to transform candidate selection and personalize neuromodulatory interventions. To truly advance the field, international collaboration is essential. Shared datasets, harmonized protocols, and multidisciplinary networks will be key to establishing validated, evidence-based algorithms for diagnosing, stratifying, and treating DoC patients.

In this evolving landscape, DBS should be reframed, not as an experimental last resort, but as a targeted, precision-guided therapy grounded in neuroanatomical reality and ethical responsibility. With the right tools, at the right time, for the right patient, the path to meaningful recovery may no longer be out of reach.

## Data Availability

The raw data supporting the conclusions of this article will be made available by the authors, without undue reservation.
